# Messenger RNA Life-Cycle in Cancer Cells: Emerging Role of Conventional and Non-Conventional RNA-Binding Proteins?

**DOI:** 10.3390/ijms19030650

**Published:** 2018-02-25

**Authors:** Lucie Coppin, Julie Leclerc, Audrey Vincent, Nicole Porchet, Pascal Pigny

**Affiliations:** 1 University of Lille, UMR-S 1172-JPARC—Jean-Pierre Aubert Research Center, F-59000 Lille, France; lucie.coppin@inserm.fr (L.C.); julie.leclerc@inserm.fr (J.L.); audrey.vincent@inserm.fr (A.V.); nicole.porchet.blg@gmail.com (N.P.); 2 Inserm, UMR-S 1172, Team “Mucins, Epithelial Differentiation and Carcinogenesis”, F-59000 Lille, Frances; 3 CHU Lille, Service de Biochimie “Hormonologie, Métabolisme-Nutrition, Oncologie”, F-59000 Lille, France

**Keywords:** mRNA life-cycle, mRNA fate, mRNA-binding proteins, mRNA-binding domains, intrinsically disordered regions, low complexity disordered regions, cytoplasmic RNA granules, DNA and RNA-binding proteins, RNA regulon, galectin-3

## Abstract

Functional specialization of cells and tissues in metazoans require specific gene expression patterns. Biological processes, thus, need precise temporal and spatial coordination of gene activity. Regulation of the fate of messenger RNA plays a crucial role in this context. In the present review, the current knowledge related to the role of RNA-binding proteins in the whole mRNA life-cycle is summarized. This field opens up a new angle for understanding the importance of the post-transcriptional control of gene expression in cancer cells. The emerging role of non-classic RNA-binding proteins is highlighted. The goal of this review is to encourage readers to view, through the mRNA life-cycle, novel aspects of the molecular basis of cancer and the potential to develop RNA-based therapies.

## 1. Introduction to the Messenger RNA (mRNA) Life-Cycle

Regulation of eukaryotic gene expression is crucial in pathophysiological responses to extracellular and intracellular signals in the context of homeostasis maintenance and cellular differentiation or in stress response and cell survival. It is the result of multiple, complex, and tightly regulated processes occurring in different cell compartments. Each process involves regulation-points, from pre-messenger RNA biosynthesis in the nucleus, to messenger RNA (mRNA) translation and, finally, to mRNA and protein catabolism in the cytoplasm. Regulation of gene expression at the mRNA level involves dynamic organization and reorganization of ribonucleoproteic structures. This requires many RNA-binding proteins controlling every step of the mRNA lifetime, e.g., nuclear maturation of transcripts, nuclear export, cytoplasmic transport and localization, stability and storage, translation, and degradation. The mRNA life-cycle can be separated into two phases, the first one being nuclear and transcriptional (also referred as RNA metabolism) and the other one being cytoplasmic and post-transcriptional. This division is artificial and is not supported by recent molecular basis. Thus, the whole process is now called the “mRNA life-cycle”. This term highlights the continuity between the different steps of mRNA fate. This continuity allows cells to quickly respond to various stimuli by regulating the expression of large subsets of messenger RNAs called RNA regulons.

## 2. Major Actors of the mRNA Life-Cycle: The RNA-Binding Proteins (RBPs)

Transcription is the first step of gene expression and involves RNA biosynthesis. From the early beginning of their transcription until they are degraded, most RNA molecules are constantly associated with proteins in cells. This rule is applicable to messenger RNAs. RNAs harbor *cis*-elements recognized by RNA-binding proteins (RBPs) in their nucleotide sequences. These RBPs can bind either to coding or non-coding sequences. They target structural specific elements that are important for RNA fate in terms of processing, transport, localization, stability, or function. Associations between RBPs and RNAs form ribonucleoproteic particles called “RNPs” or specifically called “mRNPs” when the complex involves a mRNA [1].

In the human genome, 1542 RBP genes have been identified, which corresponds to more than 7.5% of total protein-encoding genes. Their inventory has not been completed yet [2,3,4]. The RBPs they encode are able to interact with all known classes of RNAs, that is to say mRNAs (692 mRNA-binding proteins or mRBPs), but also tRNAs, pre-rRNAs, snRNAs, snoRNAs, ncRNAs, and other emerging groups of regulatory RNAs involved in translation or gene expression regulation [3]. In total, about 50% of RBPs are thought to have direct or indirect effects on the post-transcriptional regulation of gene expression and, therefore, on the intracellular fate of mRNAs [3]. 

**Figure 1:**
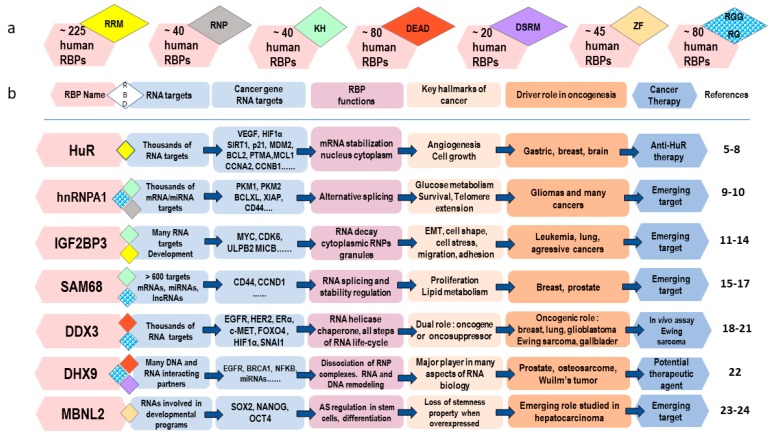
Relationships between RNA-binding domains (RBDs), RNA-binding proteins (RBPs), their RNA-binding partners, and downstream biological processes, with regards to their potential in cancer therapy. RBPs with altered expression or/and functions participate as drivers in oncogenesis and might be interesting therapeutic targets for cancer treatment [5,6,7,8,9,10,11,12,13,14,15,16,17,18,19,20,21,22,23,24]. A single RBP is able to bind several hundreds or thousands of RNA targets. Thus, the precise role and signaling pathways downstream each RNA target need to be carefully studied and elucidated to evaluate its potential in cancer therapy. More than 600 RBDs have been identified in the human genome. (**a**) Of the 692 estimated mRBPs, 405 contain an RNA recognition motif (RRM), a K homology (KH) domain, a DEAD motif (containing the amino acid sequence D-E-A-D (Asp-Glu-Ala-Asp)), a double-stranded RNA-binding motif (DSRM), a zinc-finger (ZF), or RGG/RG (i.e., Arg-Gly-Gly/Arg-Gly) motifs [2,3,4]. (**b**) Examples of RBPs representative of RBD diversity and with a potential importance in cancer therapy. The RNP domain is a RRM subclass found in heterogeneous nuclear ribonucleoproteins (hnRNP) family RBPs. As shown, many RBPs contain several RBDs. RRM, KH, DEAD, DSRM, RNP, and ZF are canonical RBDs able to adopt secondary structures with α-helices and β-sheets. RGG/RG motifs are intrinsically disordered regions (IDRs) with low ordered secondary structures, resulting in flexibility enabling them to bind with many partners. They are recognized as important emerging players in the mRNA life-cycle [2,25,26,27]. Each of the seven RBDs shown is schematically depicted with a hexagonal shape and with a specific color (as specified by the color code in (**a**)). RGG/RG motifs are highlighted by a two-color representation (blue and white).

RBPs can be classified on the structural basis of their RNA-binding domains (RBDs) (Figure 1a). RBDs are composed of 60–100 (typically 90) aminoacids. More than 600 different RBDs have been identified. Their intrinsic RNA-recognition specificity depends on their conformation, which is determined by their aminoacid sequence. The RNA-binding motif itself is short, usually between 4 to 6 nucleotides. However, it can be even shorter and restricted to a single nucleotide, as in the case of PUF domain (comprised of the Pumilio and FBF homology protein). Their binding to RNAs occur according to two modes, with single-stranded or double-stranded RNA structures [3]. Of the 692 human mRBPs, 405 mRBPs contain one or more types of mRNA-binding motifs that can be repeated either in variable numbers or in variable combinations, thus, increasing their ability to bind multiple mRNA targets in a specific manner [3].

Some examples of the most frequent RBDs are shown in Figure 1b.

Another criterion also has to be taken into consideration to understand RBP properties: it is the presence of additional protein regions working as RNA-chaperones (by helping the initially single-stranded RNA to form various secondary or tertiary structures), enzymes (such as helicase, ribonuclease, endonuclease, or telomerase), or assembling proteins [3,4]. Many of these additional protein regions are intrinsically disordered regions (IDRs, also called disordered sequences or disordered regions). They are defined as protein regions that lack any defined tertiary structure under native conditions but assume a fold in the presence of a binding partner or ligand [2]. IDRs with motifs rich in serine and arginine (S/R) and arginine and glycine (R/G) were found to contribute to RNA-binding activity. The Arg-Gly-Gly/Arg-Gly or RGG/RG motif is one of the most frequent in RBPs (Figure 1). Moreover, a large subset of RNA-binding proteins (estimated to be more than 40%) also binds double-stranded DNA, thus forming a large group of DNA-binding and RNA-binding proteins (DRBPs). These DRBPs are thought to regulate large subsets of genes involved in broad cellular processes, including transcription, translation, gene silencing, DNA damage response, telomere maintenance, apoptosis, and responses to extreme temperatures [28].

**Figure 2:**
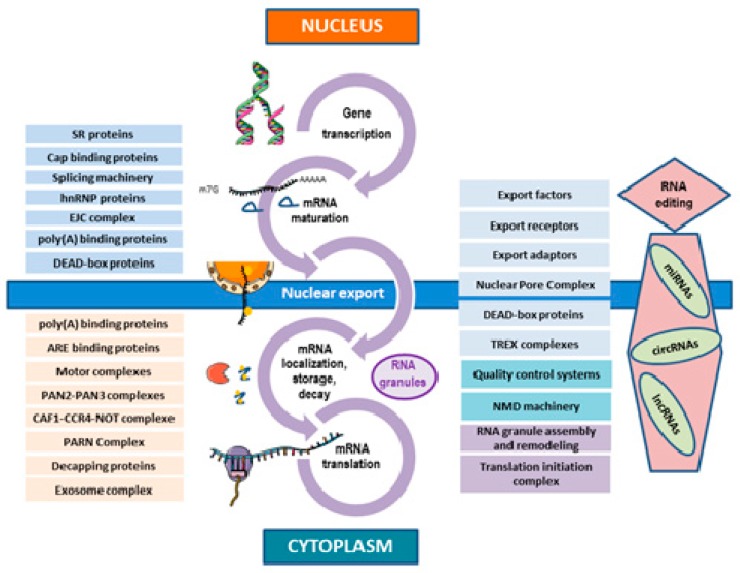
The mRNA life-cycle. This figure sums-up the different steps of the mRNA life-cycle from gene transcription to mRNA translation and degradation, occurring both in the nucleus and cytoplasm. At each step, the role of important groups of complexes, RBPs, proteins, and regulatory RNAs is shown. This figure highlights the continuity of RBP functions all along the process, even during the nuclear export. Many RBPs embedded onto mRNAs during transcription are able to cross subcellular boundaries and play a role in further steps. In blue are depicted RBPs with actions in the nucleus and factors involved in RNA nuclear export, in dark blue are systems associated with mRNA quality control, in pink are elements influencing mRNA stability and localization, and in dark purple are elements associated with mRNA fate in cytoplasm (granules and translation control). Non-coding RNAs influencing the mRNA life-cycle are also represented in this figure.

An important aspect in understanding RBP function is subcellular localization. This is either nuclear, with a putative role in splicing of pre-mRNAs, or cytoplasmic, with a supposed post-transcriptional role (Figure 2). Many RBPs are able to shuttle between the nucleus and the cytoplasm and are likely to play many roles [3,4]. Some early-RBPs remain attached to RNA during their whole lifetime, whereas others transiently bind to RNA at later stages and for specific processes only. The human antigen R (HuR) protein is a good example of an early-RNA-binding protein involved in splicing, stabilization, and translation of many mRNA species [29] (Figure 1).

**Figure 3:**
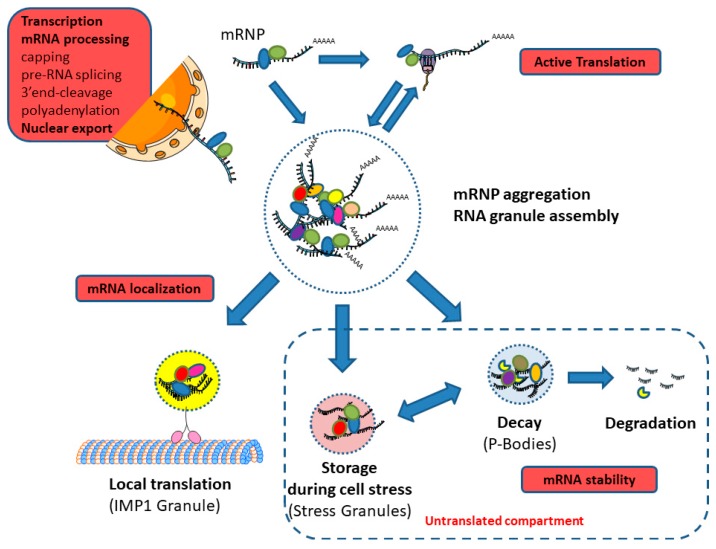
RBP functions and interplay with granule formation. This figure illustrates the different steps where RBPs control mRNA fate in the cell (symbolized by the red rectangles): gene transcription and mRNA processing in the nucleus, nuclear export, and in the cytoplasm formation, remodeling, and biological activities of RNA granules. RNA granules may contain various ribosomal subunits, translation factors, decay enzymes, helicases, scaffold proteins, and RNA-binding proteins which control the localization, stability, and translation of their RNA cargo [35]. Disordered regions of RBPs participate in the dynamic properties of granules by creating protein-protein networks, called hubs. Of the proteins in hubs, 30% are RBPs containing disordered motifs [2]. Several granule categories have been characterized, containing both common and specific granule components according to their functional role. P-bodies (PBs) are involved in mRNA decay. Stress granules (SGs) are involved in mRNA storage during stress. IMP1 (Insulin-like growth factor II mRNA-binding protein 1) granules are thought to be involved in the local translation of specific subsets of mRNAs.

Some RBPs play important roles in coordination and/or regulation between the different steps of the whole mRNA life-cycle. This occurs when coupling in time and space or a link between different molecular processes is required [30,31]. The mRNP protein content determines each mRNA fate in a spatiotemporal way. They are either immediately translated or transported into localized storage or translation areas (Figure 3) [32]. This explains why complexes between RNAs and RBPs are big and highly dynamic structures undergoing constant remodeling. This remodeling results from the versatility of competitive interactions of RBPs and other proteins into RNP complexes and RBPs being regulated by various post-translational modifications (PTMs), such as isomerization, methylation, acetylation, phosphorylation, O-GlcNAcylation (O-Linked β-*N*-acetylglucosamine modification), and ubiquitination [33,34].

All these elements highlight the complexity in summing up various functions of each of the so-called “conventional” RBPs [3]. However, an additional level of complexity comes from “unconventional” RBPs which are proteins that do not harbor any canonical direct RNA-binding sites but actively participate in the RNA life-cycle through unconventional RNA-protein interactions or/and even protein-protein interactions. They can act as a platform or scaffold that recruits many factors involved in macromolecular structure organization and remodeling of ribonucleoproteic particles. They can modify and regulate combinations of competitive binding partners in time and space, throughout the entire mRNA life-cycle. So-called “unconventional” or “non-classic” mRBPs harbor IDRs in their peptide structure, such as low complexity disordered regions (LC) containing repeated motifs rich in glycine, lysine, arginine, and even tyrosine [2]. This lack of tertiary structure gives the proteins a great flexibility to establish multiple interactions with many kinds of ligands such as RNA, DNA, other proteins, or lipids [2,27]. Moreover, the multimerization properties of some of these mRBPs promote the simultaneous recognition of several mRNAs or the interaction with several separate motifs present on the same mRNA [33]. 

As a consequence of all these features, several points can be highlighted:

- One type of mRNA may be associated with many mRBPs and each mRBP may have hundreds or thousands of different RNA targets [36].
- For each transcript, the composition of the proteins within the mRNPs can vary as it is influenced by different pathophysiological parameters.
- Use of alternative gene promoters during transcription or alternative polyadenylation sites in mRNA maturation may also constitute a source of gain or loss of *cis*-binding sequences for RBPs in the mRNA [1].

Thus, RBPs play key roles in shaping gene expression and cellular behavior throughout cell life, particularly during development. Because RBPs can be mutated or aberrantly deregulated, they are now recognized as drivers for human diseases including rare genetic diseases (i.e., developmental disorders), neurodegenerative diseases, and cancers [37,38,39]. Since 2012, large-scale mRNA interactome capture studies shedding light on RBPs and expanding their catalogues have been published on human cell cancer models such as proliferating human HeLa cells [40] or the nuclei of proliferating K562 cells [41].

Their inventory still needs to be completed through functional studies. Robust methods exist to detect and characterize the numerous binding sites of RBPs across the human transcriptome, to understand molecular mechanisms underlying their function, and, finally, construct the human protein-RNA regulatory network [42]. Future perspective lies in the production of engineering synthetic RNAs for therapeutic use.

## 3. Co-Transcriptional Processes in the mRNA Life-Cycle

Eukaryotic primary RNA transcripts newly-synthesized in the nucleus by RNA polymerase II undergo complex maturation. These maturation processes consist of the addition of a cap at their 5′ ends, a poly(A) tail at their 3′ ends, and the removal of their intronic sequences (pre-mRNA splicing). Additionally, some RNA species will undergo an editing process. These maturation processes are interconnected through different protein factors that interact with each other and with transcripts, and they control not only mRNA processing in the nucleus but also mRNA export and fate in the cytoplasm [43,44,45,46]. This specifically involves the carboxyl-terminal domain of ARN Polymerase II in which numerous serine-rich and proline-rich subdomains constitute platforms for all these interactions [1,47]. Moreover, mRNA processing in the nucleus is coupled with upstream mechanisms, such as transcription [48] and DNA damage response [49,50,51].

### 3.1. Pre-mRNA Splicing

Pre-mRNA splicing is the process by which only small segments of the primary transcripts are joined together and exported into the cytoplasm [52]. Most of the introns remain in the nucleus to be subsequently degraded or processed to give birth to many regulatory non-coding RNAs. Splicing is a highly regulated multi-step reaction carried out by the spliceosome, a huge and highly dynamic complex constantly remodeled during the process [53,54]. A spliceosome is composed of several small nuclear ribonucleoproteins (U1, U2, U4, U5, and U6 snRNP) associated with many protein cofactors. Each snRNP is itself formed by an snRNA associated with proteins [55].

The splicing process is based on the recognition by the spliceosome of short conserved sequences around each intron-exon junction and involves several successive steps. An exhaustive review describes the splicing process in detail [56]. Recently, single-RNA molecule imaging assays have been developed to observe atomic-resolution structures of the intact spliceosome at different stages of the splicing cycle [56]. A complex called the exon junction complex (EJC) is placed at the exon/exon junctions of mature mRNAs and, thus, represents a “mark” of this process. This complex is involved in further steps of the RNA life-cycle, such as mRNA nuclear export, and remains assembled on the mRNA until the first translation cycle [53,54]. 

Additional sequence elements in exons or introns can act as strong or weak positive (enhancers) or negative (silencers) regulators. They mediate the binding of splicing factors that either promote or inhibit the recognition of a given exon by the spliceosome. Thus, splicing may be "constitutive" when an exon is systematically included in the mature mRNA or, conversely, "alternative" when several different transcripts can be produced depending on the selection of the included regions. Competition between binding sites and/or between RBPs allows, from a single gene, the production of several variants resulting (for instance) from the use of 5′ and 3′ alternative splicing sites, intron retention, or exon skipping. These RBP-binding site sequences are called exonic splicing enhancers (ESE) or exonic splicing silencers (ESS) and intronic splicing enhancers (ISE) or intronic splicing silencers (ISS). These motifs are recognized by Serine/Arginine-rich (SR) proteins (which bind on enhancers) or heterogeneous nuclear ribonucleoproteins (hnRNPs) (which bind on silencers), which activate or inhibit the splicing process by affecting the spliceosome assembly [57,58]. Moreover, the presence of secondary structures in pre-mRNA influences the elongation rate of RNA polymerase II. The slower its speed, the more efficient the alternative splicing (exon retention) and vice versa (i.e., exon skipping) [59]. Several factors can modulate RNA polymerase elongation rate, such as chromatin structure (nucleosome distribution and histone modifications) or DNA methylation. Histones also recruit proteins that interact with splicing regulatory factors such as SR proteins, linking transcription to splicing steps. Moreover, cell signaling influences expression levels of splicing factors as well as their biological activities through post-translational modifications. Thus, the mitogen-activated protein kinase (MAPK) pathway regulates the alternative splicing of *CD44* mRNA through Sam68 (i.e., the Src-associated substrate in Mitosis, 68 kDa) splicing factor phosphorylation (Figure 1). Furthermore, subcellular localization of splicing factors or regulators (nucleocytoplasmic distribution, transport, and trafficking) can influence this process [57]. The MAPK, phosphatidylinositol 3 (PI3) kinase (PI3K), and wingless (Wnt) pathways, as well as signals from the microenvironment, influence the subcellular localization of the SR proteins and hnRNPs and their activity [60]. 

Mutations occurring in the nucleotide sequence of splicing sites, in splicing factors, and/or in RBP partners may lead to the deregulations or abnormalities in mRNA splicing at the origin of many disorders, including cancer, and have been described in exhaustive reviews [61,62]. In cancer cells, oncogenic mutations affect splicing factor binding, nuclear translocation, expression, or proteosomal degradation of RBPs. Changes in splicing programs have strong effects on cell proliferation, invasion, cell survival, and angiogenesis [60]. Among all mutations, those affecting genes directly encoding components or associated factors of the U1, U2, U4, U5, and U6 snRNP have strong effects and lead to many mis-spliced mRNAs. Selective depletion of the serine and arginine-rich splicing factor 1 (SRSF1), a SR protein involved in binding U1 snRNP to a 5-splice site-containing pre-mRNA, was reported to affect 498 splicing events [63]. 

SR and hnRNP RBP families are important regulators of apoptosis. Expression of SRSF1, SRSF2, SRSF3, SRSF5, SRSF6, hnRNPA1, hnRNPA2, hnRNPB1, hnRNPH, and hnRNPK is frequently disturbed in cancers, which leads to impaired alternative splicing of key apoptotic mRNA targets. This alters the balance between pro-apoptotic and anti-apoptotic factors facilitating cancer cell survival [64].

### 3.2. Cap and Poly(A) Tail Addition

At the early beginnings of transcription, as soon as a pre-mRNA molecule reaches a size of 20–30 nt, a cap is added at its 5′ end to protect it from degradation by the 5′–3′ exonucleases. This cap consists of a 7-methylguanosine attached to the first nucleotide of the mRNA by action of three enzymes [47]. It is involved in splicing through the cap-binding complex (CBC) which recruits U1snRNP and U6snRNP. It is also required for nuclear mRNA export and translation [47]. For novel findings in RNA capping and the issues these findings raise, see reference [65].

mRNAs harbor a simple structure called the poly(A) tail at their 3′ end, which is a 3′ terminal polyadenosine tract (of 200 to 250 adenosines on average) added after the cleavage of transcripts downstream a polyadenylation site (AAUAAA motif). A poly(A) tail is recognized by poly(A) binding proteins (PABPs), which protect RNA from cleavage by 3′–5′ exonucleases and deadenylases. The poly(A) tail is shortened over time and is finally removed at the end of mRNA life-cycle. Thus, the poly(A) length has a dramatic impact on multiple aspects of mRNA fate. It participates in mRNA nuclear export, stability, and translation [47] and might be seen as an important parameter of post-transcriptional control of gene expression during development, differentiation, and in cancer [66,67,68]. Several mRNAs can be generated from a single gene through the use of alternative polyadenylation (APA) signals in their 3′UTR region, leading to differential temporal and spatial regulation of these mRNAs and, thus, modulation of gene expression [69].

Altered 3′ end processing leads to many disorders, recently reviewed in reference [69]. In cancer cells, the use of a proximal polyadenylation site increases mRNA stability of some targets and, thus, leads to the production of proteins that promote some properties of cancer cells. For instance, modified APA patterns have been reported during the progression of colorectal cancer, affecting three genes: dermokine (*DMKN*), pyridoxal kinase (*PDXK*), and peptidylpropyl isomerase E (*PPIE*) [69].

## 4. Post-Transcriptional Processes in the mRNA Life-Cycle

### 4.1. Nuclear Export

Only fully mature mRNA molecules can be exported from the nucleus to the cytoplasm. Proteins involved in this transport are recruited during RNA nuclear maturation [70]. Aberrant or non-fully mature products are detected by quality control systems and degraded in the nucleus. 

mRNP nuclear export requires proteins acting as adaptors and receptors. Many different adaptors are present in each mRNP at the time of release from the gene and two main export receptors, nuclear export factor 1 (NXF1) and chromosomal maintenance 1 (CRM1, also called exportin 1), interact with different sets of export adaptors [71]. This nuclear transport can use different pathways and can be selective for some mRNA species, thus acting as an additional level of gene expression regulation [72]. 

Nuclear export of mRNPs is carried out through nuclear pores embedded in the nuclear envelope and requires the presence of the NXF1 protein and its co-factor, NXT1 (nuclear transport factor 2 like export factor 1) or the presence of the export receptor CRM1, according to length of the transcripts [72]. Nuclear pore complex (NPC) permeability is controlled by FG-nucleoporins, rich in phenylalanine and glycine (F and G), which are recognized by the NXF1 and CRM1 factors [72]. Moreover, transcription-export (TREX) complexes are recruited at the 5′ cap via the CBC and escort mRNAs and their associated proteins up to the NPC [73]. When mRNP reaches the NPC cytoplasmic side, DEAD-box RBPs bind to mRNPs, inducing a change in their conformation (a mechanism that requires ATP) and resulting in a remodeling of their protein content. For instance, the CBC complex is replaced by the eIF4E translation initiation factor [70].

Cells are able to regulate the nucleocytoplasmic transport of specific mRNA species, introducing an additional level of control for gene expression. Intriguingly, messenger RNAs that encode functionally-related proteins requiring a temporally coordinated expression might be exported, localized, and translated together [74]. This phenomenon has been described as the RNA regulon model [75]. Some key factors in mRNA export are regulated by proliferative signaling, thus suggesting it could be involved in cancers. Mutations responsible for aberrant mRNA export mechanisms in diseases have been reported and they lead to nuclear accumulation of mRNAs [76,77]. Deregulation of mRNA export key factor expression occurs in many types of cancer cells due to proliferative signaling involving c-Myc or cyclin D1 through the increase of eiF4E and CRM1 protein levels [75,77,78].

The mRNP composition is remodeled close to the nuclear envelope before cytoplasmic release but also after release. The new cytoplasmic mRNP may gain additional factors (i.e., RBPs, non-coding RNAs, and microRNAs) that control its mobility, further localization, and translation (Figure 3). In the cytoplasm, mRBPs may directly or indirectly bind to motor proteins (i.e., kinesins, dyneins, and myosins) to form high molecular weight mRNP motor complexes [79].

### 4.2. mRNA Cytoplasmic Degradation

In eukaryotes, intracytoplasmic mRNAs can be degraded through two main pathways, both starting with deadenylation. Deadenylation takes place in two successive steps involving, firstly, the PAN2-PAN3 complex (poly(A) nuclease 2 and 3) and, secondly, the CAF1-CCR4-NOT complex or the deadenylation complex PARN (poly(A)-specific ribonuclease) [80]. Once deadenylated, mRNAs can either [81]:

- be degraded in the 3′ to 5′ direction by the exosome, a multiprotein complex that recruits RNAses and cofactors, the activity of which is regulated by the SKI (Sloan-Kettering Institute) complex; or
- be decapped by decapping protein 2 (DCP2) and its co-activator, DCP1. This step is followed by an exonucleolytic degradation in the 5′ to 3′ direction performed by the exoribonuclease, XRN1.

Most transcripts are degraded by this deadenylation-dependent pathway. However, alternative pathways can be used, such as an endonucleolytic cleavage mediated by inositol-requiring enzyme 1 (IRE1) [82].

Several mRNA quality controls allow only mature mRNAs without any abnormalities to be accessible to the translation machinery. Indeed, aberration detection during translation leads to the elimination of these mRNAs by specific surveillance or degradation pathways such as nonsense mediated decay (NMD), non-EJC-dependent NMD mechanism, no-go decay, or non-stop decay [53,82,83].

Genetic disorders may be caused by defects in genes involved in mRNA surveillance pathways. NMD contributes to cancer pathogenesis through overexpression or loss of function of factors involved in this mRNA quality control. For instance, mutations in the *UPF1* gene have been described in pancreatic adenosquamous carcinomas. Mis-splicing leads to the loss of UPF1 function in NMD and, thus, the accumulation of mRNAs with premature termination codons impairing important functions, such as those of the tumor suppressor TP53 [84]. However, transcriptome-wide profiling studies of NMD deficient cells lead us to consider that NMD also constitutes an additional post-transcriptional layer of gene expression control involved in the regulation of many biological pathways. Thus, NMD contributes to regulate cellular stress response and homeostasis of splicing regulators and NMD factors, and these new roles might be impaired in cancer cells [85].

### 4.3. Factors Modulating mRNA Stability

The mRNA level in a cell at a given time point is the result of the balance between newly-synthetized mRNA molecules (defined by the transcription rate) and degraded mRNA molecules (defined by the degradation rate). Many *cis*-elements are involved in mRNA stability. In most cases, they are localized in the untranslated transcribed region (UTR), especially in 3′UTR. We will focus on the influence of the poly(A) tail, microRNA-binding sites, and two *cis*-elements—AU rich elements (AREs) and the CA repeat element (CARE)—on mRNA stability. 

#### 4.3.1. Poly(A) Tail and PABP

The poly(A) tail is bound by a family of poly(A) binding proteins (PABPs). There are four PABPs, one being nuclear (PABPN1) (with a high affinity for nascent poly(A) chains) and the other three being cytoplasmic (PABPC1, PABPC3, PABPC4). Of these, PABPC1 is the best characterized. In the nucleus, PABPN1, through its binding to the first A nucleotides added to the poly(A) tail, stimulates the activity of the poly(A) polymerase. This allows the elongation of the poly(A) tail. It is also involved in the export of mRNAs [86]. Cytosolic PABPC influences the stability and translation of mRNAs through their binding to the poly(A) tail.

Regarding mRNA stability, PABPs protect the poly(A) tail from deadenylation by preventing its access to deadenylases. They also protect the 5′ end of mRNA from decapping through the formation of a PABP-eIF4G-eIF4E complex [87]. PABPs play many roles. They regulate the recruitment of endonucleases and interact with ARE-binding proteins. They also interact with eIF4G to create a loop between the 5′ and 3′ ends of the mRNAs, which stimulates the assembly of the translation initiation complex. PABPs can also interact with eukaryotic release factor 3 (eRF3), a translation termination factor. This interaction regulates translation by increasing ribosome recycling to the 5′ end of mRNAs in order to initiate several cycles of translation [87]. This interaction is also involved in the control of mRNA degradation. Binding affinity between eRF3 and PABP has been shown to be reduced in patient families suffering from gastric and breast cancer susceptibility [88].

#### 4.3.2. Non-Coding RNAs and RNA Editing

Only 2% of the mammalian genome encodes mRNAs, and most of the transcripts are non-coding RNAs. Non-coding RNAs play critical roles as transcriptional and post-transcriptional regulators of mRNA. Among them, microRNAs are well recognized for their role in cancer. MicroRNAs (miRNAs or miRs) are small non-coding RNAs of about 21 nucleotides in length incorporated into a ribonucleoprotein complex called a miRNA-induced silencing complex (miRISC). Within this complex, the major RBPs are the GW182 (glycine-tryptophan protein, 182 kDa) proteins and the Argonaute (Ago) family proteins. Among Ago proteins, only Ago2 has a RNAseH-like PIWI (P-element Induced WImpy) domain activity allowing for the cleavage of the target mRNAs [89].

The specificity of mRNA target recognition by microRNAs is ensured by their seed sequence (corresponding to nucleotides 2 to 8 in the 5′ region of microRNA). They mostly interact with mRNA 3′UTR regions, but binding sites are also found in the 5′UTR regions and even in coding regions. Pairing between the microRNA and mRNA targets determines the mRNA fate with two modes: mRNA degradation or silencing by preventing translation [90]. mRNA degradation controlled by microRNAs requires cytoplasmic components found in cytoplasmic granules called processing-bodies (P-bodies or PBs). Interestingly, microRNA binding to the 5′UTR regions of genes is associated with translation activation rather than repression [91]. In addition, microRNAs can regulate gene expression based on other ways. They inhibit gene expression at the transcriptional level through direct DNA binding. They can also bind to RBDs in RBPs, which prevents the binding of these RBPs to their mRNA targets, thus modulating their functions [92].

MicroRNAs target more than 50% of human protein-coding genes and, hence, play a central role in various pathological conditions, including neurodegenerative diseases and cancers. Since their discovery in 1993, many articles described a role played by microRNAs in every step on the road to cancer biogenesis and progression. Today, there are numerous examples of microRNAs involved in the hallmark of cancer. Down-regulation or up-regulation of microRNA expression contributes to cancer driving steps, as well as mutations or polymorphisms in the microRNA nucleotide sequence, microRNA biogenesis related genes, or target binding sites [93]. For instance, the let-7 family of microRNAs (highly conserved in metazoans) functions as a master regulator of development and differentiation, with many important target genes involved in cell cycle regulation, such as *MYC, DROSHA, IMP1* (insulin-like growth factor 2 mRNA-binding protein 1), or *HMGA2* (an oncofetal and pluripotency factor). In papillary thyroid carcinomas, miR-let-7a is downregulated, and one of its targets, *AKT2,* is thus overexpressed in these tumors. This enhances tumor migration, invasion, and growth [94]. In various diseases (including cancers), deregulation of microRNAs is commonly associated with deregulation of splicing factors such as SR proteins and hnRNPs [95]. 

It is important to mention the role of a wide emerging group of endogenous regulatory ncRNAs, called circular RNAs (CircRNAs). Generated either from exons or introns during splicing or the action of RBPs, circRNAs are thought to play important roles in gene expression regulation as miRNA-sponges, RBP-sponges, and protein/peptide translators [96]. Aberrantly expressed in various human diseases such as cancers, they are considered emerging diagnostic and therapeutic biomarkers [97,98].

Previously misconstrued as artifacts of transcription or chromatin remodeling, long non-coding RNAs (lncRNAs) are non-coding RNA transcripts more than 200 nucleotides in length. They are involved in many cellular processes, such as gene imprinting, differentiation, and development, or in the antiviral response. Among the great variety of mechanisms reported, some maintain the structure of nuclear speckles, interfere with regulation of transcription via interactions with the chromatin complexes, or interfere with splicing regulation in the nucleus. Others regulate mRNA decay in the cytoplasm or translation in the polysomes. Despite the large number of lncRNAs in the human genome, only a few have been well-characterized so far. In cancers, mutations causing deregulation of some lncRNA genes which contribute to cancer signaling pathways have been reported. However, functional studies using in vivo models have yet to be conducted to reveal the cancer relevance of lncRNAs in apoptosis, survival, metastasis, or metabolism [99].

Adenosine deamination in the transcriptome results in the formation of inosine. This process is called A-to-I RNA editing and is catalyzed by adenosine deaminase acting on RNA (ADAR) enzymes (e.g., ADAR1, ADAR2). The critical role of RNA editing has emerged in many aspects of RNA metabolism, including mRNA stability, splicing, nuclear export, and localization, as well as in the recoding of proteins. A-to-I editing in the seed region of microRNAs leads to functional consequences. lncRNA binding to precursor messenger RNA can create a double stranded-RNA region, a prerequisite for ADAR1/2-mediated RNA editing. ADAR1 editing of inverted Alu sequences present in the 3′UTR of a transcript may result in the recruitment of HuR, which stabilizes transcripts. Several studies have explored the role of ADAR enzymes in disease. ADAR2 is involved in the pathogenesis of brain tumors and is severely hindered in patients with glioblastoma. This deficiency is associated with tumor progression [100].

#### 4.3.3. Cis-Elements (AU Rich Elements and CA Repeat Elements) 

Among the *cis*-elements known to influence mRNAs stability, AREs are among the best characterized. AREs are adenine and uracil-rich motifs found in the 3′UTR region of mRNAs with short lifetime encoding proteins that have transient expressions, such as cyclins and growth factors. However, the role of AREs on mRNA stability depends on the type of ARE-binding proteins (ARE-BP) binding to them. For instance, tristetrapolin (TTP) promotes rapid degradation of *GM-CSF* (Granulocyte-macrophage colony-stimulating factor) mRNAs, while HuR stabilizes *VEGF* transcripts [81]. When AREs are destabilizers, they target mRNAs to degradation by a mechanism dependent on deadenylation and exosome degradation machinery [101]. mRNA stabilization mediated by ARE-BPs can be due to the competitive binding of a destabilizing factor on the transcript [82]. Moreover, post-translational modifications of ARE-BPs modulate their affinity for AREs or their cellular localization [102]. The effect of ARE-BPs on mRNA can also be modulated by microRNAs that bind to the same transcript in a cooperative or competitive manner [103]. ARE-mediated regulation is specific to subsets of transcripts harboring the same ARE motif with related functions. ARE-BPs control translation through two modes: (1) degradation of the poly(A) tail by releasing PABPs and (2) binding to eIF4E, which prevents its binding to eIF4G. Moreover, in stressed cells, some ARE-BPs guide RNAs to cytoplasmic granules called stress granules (SGs), in which ribosomes on mRNAs are stalled at the initiation of translation (Figure 3) [101].

Downregulation of ARE-BPs is observed in cancers, leading to stabilization of mRNA targets involved in the cell cycle (cyclin D1, for example), in angiogenesis (VEGF, for example), or in apoptotic resistance (BCL2, for example). Thus, TTP downregulation in breast cancer is associated with a negative prognostic factor [104].

Besides AREs, CA repeat elements are other *cis*-elements modulating mRNA stability. For example, in hypoxic conditions, hnRNP stabilizes *VEGF* mRNA through binding to a CARE [105]. This interaction prevents the inhibitory activity of miR-297 and miR-299 (in normoxic conditions) by blocking the access of the miRISC complex that binds the same element. 

### 4.4. Cytoplasmic Granules

Control of translation and cytoplasmic mRNA degradation can be achieved through transcript storage into cytoplasmic higher-order structures of ribonucleoprotein (RNP) called granules [106]. Cytoplasmic RNA granules are membraneless intracellular organelles which may contain many components such as ribosomal subunits, translation factors, decay enzymes, helicases, scaffold proteins, and RNA-binding proteins. They control the localization, stability, and translation of their RNA cargo [35]. Granules interact with the cytoskeleton, which plays an important role in their dynamic properties [107].

During stress-induced mRNA release from polysomes, transcripts can be stored and concentrated in granules where translation is inhibited [108]. This concerns mainly PBs and SGs, according to their molecular composition [74]. These granules modulate mRNA translation (in SGs) or degradation (in PBs) (Figure 3) [106]. These two types of assemblies contain defined populations of RNA (including mRNA) and proteins, such as enzymes, protein-binding proteins, and RNA-binding proteins. Some of these components are common and others are specific to each type. The hallmark of SGs is the presence of stalled 48S translation initiation complexes and stress-dependent RNA-binding factor, whereas PBs contain mRNA machinery decay and factors of NMD [109]. SGs and PBs interact together and mRNAs can pass from one type of granule to another. When stress is resolved, mRNAs can either return to the active pool of translation or be degraded. Some proteins found in cytoplasmic bodies are characterized by the presence of LC regions in their peptide structures, which is thought to be important for driving assembly or disassembly of these highly dynamic structures [26]. For example, interactions between defined subpopulations of RNAs create networks that are essential for regulatory mechanisms of gene expression in stem cells [110].

Other categories of RNA granules have been discovered in germ cells or in neurons (i.e., HuR granules) [111]. Interestingly, one study described IMP1 (Insulin-like growth factor II mRNA-binding protein 1) granules that contain translationally repressed mRNA encoding proteins involved in endoplasmic reticulum quality control, ubiquitin metabolism, and secretory pathways. These granules would, therefore, constitute post-translational mRNA operons, allowing a coordinated and local production of proteins encoded by the mRNA they store (Figure 3) [112]. Moreover, transport granules direct mRNA to specific subcellular sites, enabling localized translation in cells. These granules formed by RNP oligomerization through protein–protein interactions are also associated with motor proteins, allowing their mobility along cytoskeleton elements [113]. Translation is repressed during transport [114].

Diseases involving deregulation of the RNA granule assembly or disassembly/clearance have been recently reported, showing a broad spectrum of phenotypes. Among them, most are rare genetic diseases, but a subclass of pediatric cancer (medulloblastoma) has also been described [115]. It will be important to understand molecular and cellular mechanisms behind these diseases to conceive therapeutic approaches targeting this aspect of the RNA life-cycle. 

## 5. Interplay between the Different Stages of mRNA Life-Cycle and Coupling to Other Vital Cellular Processes

Groups of mRNAs encoding functionally related proteins are coordinately regulated by one or more specific RBPs as post-transcriptional (PT) regulons, leading to proper expression and stoichiometry of proteins and metabolites in cell machineries, complexes, or pathways. Modulation of RBP activities though signaling pathways is a common way to control PT regulons through factors able to stimulate or repress both mRNA synthesis and decay, as well as coupling their expression to cell responses to external stimuli. For example, the signaling factor MAPK p38 (activated by stress and inflammation) is able to regulate TTP activity. TTP is a typical RBP able to regulate the stability of a large number of mRNA species [46].

The coupling between transcription and downstream RNA processing involves the RNA polymerase II and splicing factors. Coupling between transcription, mRNA export, mRNA localization, and translation involves many RNA-BPs embedded onto mRNAs during transcription and able to cross subcellular boundaries. Termination of mRNA export is linked to translation and mRNA turnover [116,117]. Splicing factors are able to control translation regulation through the control of microRNA biogenesis [95]. Moreover, it has recently emerged that RNA processing and DNA damage response (DDR) are able to influence each other through the role of some factors involved in alternative splicing [49,50]. Thus, it clearly appears that an additional layer of complexity in the regulation of gene expression has emerged, linked to the presence of many factors packaged or embedded onto RNPs. Many of these factors are yet unknown or, if identified, have unknown functions. Novel findings in the field of RBPs are expected from proteins harboring IDRs, especially from a subclass of these called LC sequences [2,27,49,50]. LC sequences (composed by up to hundreds of repetitions of one or several amino acids) exhibit frequent mutations in cancers and play a significant role in many basic cellular functions such as mRNA translational repression, DNA damage signaling, transcription, regulation of apoptosis, and pre-mRNA splicing [25,49,50]. Strikingly, both known and novel RBPs are significantly enriched in disordered regions compared to the total human proteome. Approximately 20% of the identified mammalian RBPs are disordered by over 80%. Another important role for disordered regions of RBPs is to contain RNA in membraneless RNA granules where it can be stored or processed [26]. To illustrate this point, one can summarize several independent recent publications on three gene families—galectins, SR proteins, and hnRNPs—with functional potential interaction between classic and non-classic RBPs.

**Figure 4:**
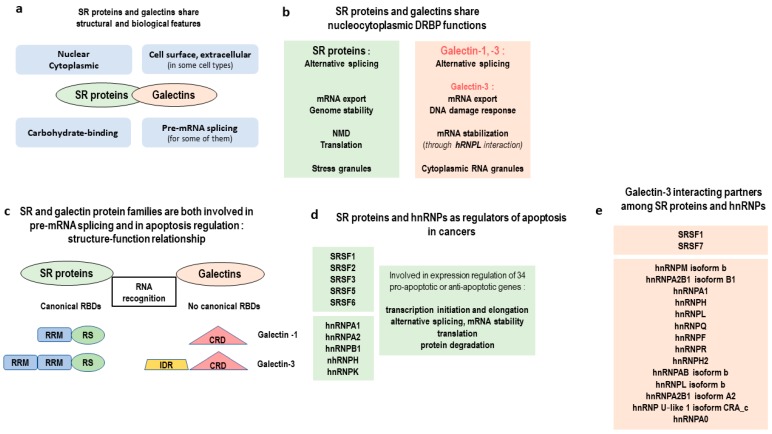
Towards a network of RBPs involved in apoptosis regulation. (**a**) As early as 2010, Haudek et al. [118] highlighted four striking similarities (in blue rectangles) between the general features of two protein families, SR (serine arginine rich) proteins (previously known as splicing factors) and galectins (previously known as animal lectins binding β-galactoside motifs found in many glycoproteins and glycolipids). They share a large intracellular and extracellular distribution, potentially indicating their involvement in large cellular processes with broad functions. (**b**) SR proteins and two members of the galectin family, galectin-1 and galectin-3, are expressed in the nucleus and involved in pre-mRNA splicing. However, SR proteins and galectin-3 share some other striking features, such as shuttling between the nucleus and cytoplasm and involvement in genome stability [120,122,123,124,125,126]. The RBP role of galectin-3 is mediated through the hnRNPL interaction, as shown by Coppin et al. [120]. (**c**) SR proteins harbor two RBDs, RRM (RNA recognition motif, a canonical RBD) and SR (an RBD with some Intrinsically Disordered Regions (IDRs)). Galectin-1 and galectin-3 harbor a carbohydrate-recognizing domain (CRD) but no RBD. However, galectin-3 harbors an IDR. (**d**) SR proteins and hnRNPs regulate the alternative splicing of pre-mRNAs involved in the expression of pro-apoptotic or anti-apoptotic variants, thus influencing their balance in cancer cells. Besides their role in alternative splicing, they are also involved in all stages of the expression regulation of 34 target genes involved in apoptosis regulation [64]. (**e**) 84 nuclear binding partners of galectin-3 were identified by Fritsch et al. through galactose interaction assay [121], making them interesting targets in studying new galectin-3 functions. In this report, two Serine/Arginine-rich splicing factors (SRSFs) and 14 hnRNPs, including hnRNP-L, are found. Thus, galectin-3 is thought to act as a major player in large cellular processes, including mitosis and DNA damage response, transcription and translation, apoptosis, and responses to stress [121].

**Figure 5:**
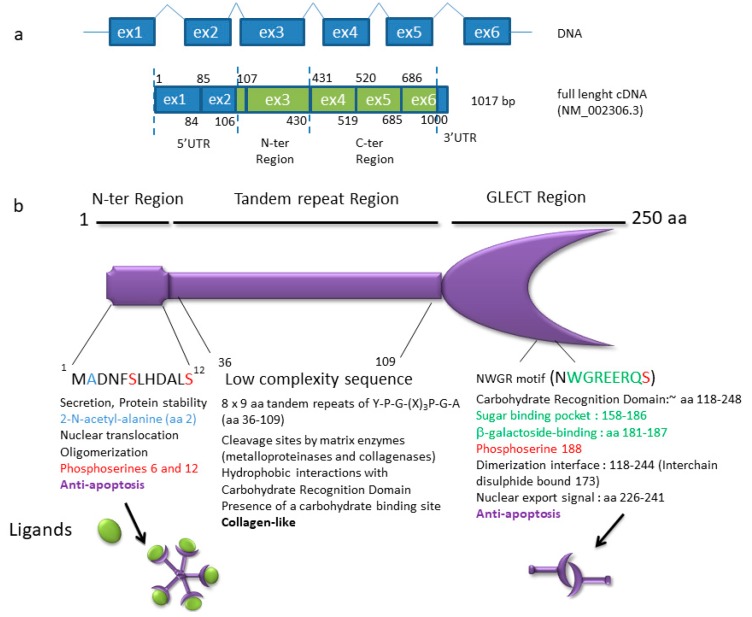
Galectin-3 is an example of a non-classic DNA-binding and RNA-binding protein (DRBP) harboring a large IDR. (**a**) The *LGALS3* gene maps to 14q21-2. Six exons are found in full-length cDNA. In the bottom schema, protein coding sequences are highlighted in green, whereas untranslated exons are depicted in blue. Three alternative spliced variants encode three proteins which are 250 (full-length, shown in b), 223, and 88 amino acids (aa) (not shown), respectively. Four alternative spliced transcripts (not shown) are non-coding RNAs. (**b**) Functional domains of human galectin-3 (comprised of 250 aa). Galectin-3 is composed of three major domains [127,128]. Two of these domains—the short 12 aa *N*-terminal region and the 130 aa globular *C*-terminal domain (118–248 aa) containing the CRD with the carbohydrate binding site (181–187 aa) [119,127] are involved in apoptosis regulation. Galectins are a large family of proteins with a galactose-lectin region (GLECT) containing a common CRD able to bind many glycoprotein and glycolipid targets with β-galactoside motifs. They regulate cell death both intracellularly and extracellularly and have other relatively broad functions. Galectin-3 is the only galectin with both anti-apoptotic and pro-apoptotic activities, according to cell types. This is also the only galectin to harbor a long IDR in its structure, between the *N*- terminal and *C*-terminal domains [129]. The galectin-3 IDR is a LC sequence harboring a stretch of repetitions of three aa (proline, glycine, and tyrosine) with a 9 aa collagen-like consensus sequence: Y-P-G-(X)_3_-P-G-A. This domain (with approximately from 20 to 100 amino acids) interacts with itself and with a part of the CRD not involved in carbohydrate recognition (approximately 200–220 amino acids), forming a fuzzy complex via intermolecular and intramolecular interactions, mainly through hydrophobicity [119]. A new carbohydrate binding site was recently identified in the LC [130]. Like many RBPs, galectin-3 harbors phosphorylable serines (three serines highlighted in red) and is able to constitute multimers (pentamers). The β-galactoside binding motif is in green. Galectin-3 is involved in the mRNA life-cycle through different ways: through binding with the ribonucleoprotein U1 of the spliceosome [131,132], through stabilizing mRNA (through hnRNP-L interaction), and through mRNA nuclear export and storage in cytoplasmic granules [120]. Galectin-3 also acts as a player in the DNA damage response through its interaction with BARD1 in a large complex involving a BRCA1/BARD1 heterodimer [126]. Galectin-3 interacts in an O-GlcNAcylation-dependent manner with a key mitotic regulator, the nuclear mitotic apparatus protein (NuMA), which is required for the establishment and the cohesion of the mitotic spindle [125]. The subdomains or motifs involved in DRBP functions which are newly attributed to galectin-3 have not been identified yet.

In 2010, Haudek et al. highlighted some intriguing structural and biological features shared by galectins and SR proteins (Figure 4a) [118]. They exhibit an expression in several subcellular compartments, are able to bind sugars, and are involved in mRNA alternative splicing (Figure 4b,c). In SR proteins, the canonical RRM domain is considered responsible for RNA-recognition specificity in mRNA alternative splicing, but in galectin-1 and galectin-3 no RBDs are characterized (Figure 4c, Figure 5a,b). It has been shown that, in SR proteins, a second typical domain of this family, called the RS domain, exhibits similar properties to IDPs. This domain is able to interact with other splicing enhancer factors than those binding to RRM and promotes pre-spliceosome assembly formation [27]. In galectin-3, besides the carbohydrate domain (CRD) considered responsible for the major functions of this galectin, a long *N*-terminal LC sequence is found which interacts with the CRD [119]. Experiments performed in our laboratory showed that, despite the absence of a canonical RBD, galectin-3 is a new non-classic RBP able to stabilize mRNAs without direct interaction with an RNA target. It does this, instead, through an hnRNP-L interaction. Moreover, this work demonstrates that galectin-3 is involved in RNA nuclear export and storage in cytoplasmic granules of *MUC4* mRNA [120]. Many questions arise now on a possible common role played by SR proteins, hnRNPs, and galectin-3 in the deregulation of apoptotic genes in cancers. A recent review highlights the complex and dual role of SR proteins and hnRNPs, not only on alternative splicing but also on mRNA stability, translation, and protein degradation of 34 genes involved in apoptosis regulation (Figure 4d) [64]. On the other hand, works by Fritsch et al. identified protein partners of galectin-3 in the nucleus. In this report, 14 galectin-3 binding-partners were hnRNPs and two were SR proteins (Figure 4e) [121]. Together, this data expands the relevance of a network of RBPs involved in apoptosis regulation.

Characterization of new factors acting on mRNAs and their functional relationship with canonical RBPs, as well as those regulating or coordinating all complex stages of the whole mRNA life-cycle, is necessary to conceive new RBP-based anticancer therapies. 

## 6. Therapeutic Approaches Targeting RBPs and Related mRNA

Targeting the mRNA life-cycle appears to be a relevant strategy in developing new anti-cancer therapies. As previously described, many actors involved in the mRNA life-cycle can be targeted, such as microRNAs, RBPs, and RBP expression protein regulators. Alternative splicing can also be considered, as recently reviewed by Song et al. [133].

### 6.1. Which Targets?

An increasing number of targets, including RBPs such as HuR, hnRNPA1, IGFBP3, Sam68, DDX3, DHX9, and MBNL2, are under evaluation for the development of new cancer treatments [5,6,7,8,9,10,11,12,13,14,15,16,17,18,19,20,21,22,23,24].

It is interesting to focus on multi-functional cancer players such as the Myc protein. Myc is well known to function as a transcription factor which is able to positively or negatively regulate the transcription of sets of genes mainly involved in proliferation, cell growth, reprogramming, and RNA biogenesis. However, Myc is also a regulator of many post-transcriptional mechanisms. It promotes cap addition for its target genes, upregulates several components of the alternative and constitutive splicing machinery, and it indirectly regulates RNA degradation by modulating the expression of AU-binding proteins (AUBPs) and components of the exosome machinery. On the other hand, Myc inhibits the NMD pathway and upregulates the transcription of ribosomal RNAs by RNA polymerase I and III, as well as enhances pre-rRNA processing and rRNA post transcriptional processing. Myc also regulates the translation of many targets through the modulation of several microRNAs’ maturation. Myc also controls the expression of the hnRNP protein family [9]. All these functions of RNA processing make the *MYC* gene a target for new cancer therapy [134]. 

MicroRNAs constitute promising elements for cancer diagnosis or cancer patient stratification. In vitro and in vivo studies have also shown that, in cancer, microRNAs such as let-7, miR-10b, miR-21, miR-34, miR-155, and miR-221 are promising targets in developing miRNA-based therapy for human malignancies [135,136,137,138,139,140].

### 6.2. Which Tools?

These last few years, new therapeutic tools have been developed to target the mRNA life-cycle. Synthetic RNA oligonucleotides hybridizing to specific microRNAs are, for instance, tested for kidney injury and fibrosis treatments through miR-21 targeting. miR-21 overexpression contributes to the pathogenesis of the disease through inhibition of mitochondrial biogenesis and functions [141]. A recent preclinical study describes the use of an anti-sense oligonucleotide (ASO) targeting *MALAT1* lncRNA in a mouse breast cancer model [142,143,144]. RNAi strategies are also being developed in cancer to inhibit activated oncogenes contributing to tumor growth. The main challenge of this strategy is to specifically target the affected organ. To achieve this purpose, nanoparticles are being developed and investigated in vitro before clinical applications. For instance, gold nanoparticles are tested for microRNA delivery in human multiple myeloma [145]. Recent studies have demonstrated that microRNAs act as hormones because extracellular microRNAs are able to shuttle between cancer cells and their neighboring cells. They are also located in extracellular vesicles such as exosomes which contribute to cell–cell communication. These exosomal microRNAs are involved in tumor growth enhancement or limitation according to their effects. Strategies targeting these exosomal microRNAs rather than cancer cells are under investigation due to difficulties to target organs specifically [146,147].

Since RNA mis-splicing plays key roles in cancers, it should be of interest to manipulate splicing of specific RNA targets to restore normal isoforms in cancers. However, there is currently limited technology targeting RNA splicing. The most commonly used method is based on ASOs that pair with splicing sites or splicing regulatory *cis*-elements. Due to difficulties for ASOs in reaching the nucleus, the CRISPR-Cas9 system could alternatively be used to delete targeted exons (i.e., genome editing) or to inhibit gene expression (i.e., epigenome editing). Engineered RNA-binding domains of splicing factors that bind to specific *cis*-elements, in order to modulate alternative splicing, constitute another way to achieve this purpose. This last strategy has demonstrated efficiency in increasing tumor cell apoptosis and the sensibility of the tumor cell to chemotherapy [148]. For instance, in breast cancer, alternative splicing of the estrogen and progesterone receptors are responsible for the inhibition of the response to hormone therapies in patients with a positive status for these receptors [149]. A recent review details therapeutic modulation of splicing in glioblastoma [150].

The direct use of RNA constitutes another way to take advantage of mRNA metabolism. A recent study describes the use of a RNA-lipoplexes vaccine in mice to specifically deliver RNA-encoding tumor proteins to dendritic cells, instead of directly exposing these cells to tumor antigens. This strategy was able to prevent tumor development when the treatment was administered before the tumor cell grafts. Successful results were also observed to have a therapeutic purpose since tumors were reduced in size or disappeared when the vaccine was given to mice after tumor cell grafts [151]. 

As we previously described, Myc is a key factor in carcinogenesis. To target it, several strategies have been developed, such as small inhibitors acting as transcriptional repressors of the *MYC* gene or ASOs. Dominant negative inhibitors complexing with the Myc protein have also been developed to block its interaction with binding partners and, thus, inhibit its activity [5,152]. 

In the same way, tools targeting key RBPs in the mRNA life-cycle are of interest. For instance, strategies disrupting HuR binding to mRNA with chemical compounds are able to inhibit HuR activity, as described by Wu et al. [8] (Figure 1). 

RNA-binding proteomes show the important place of IDRs in the ever-growing field of the RNA-interactome. The important role of IDRs in the dynamics of RNA ultrastructures, such as granules, is largely unexplored today. Nevertheless, because IDRs in granules are thought to regulate many aspects of RNA stability and translation during stress, they should be the subject of more systematic functional studies which could lead to therapeutic innovations, particularly in oncology.

## 7. Conclusions and Take-Home Messages

Until recently, the number of human RBPs, initially identified through their canonical RNA binding domains, was estimated to be around 500. Thanks to new methods of genome-wide RNA target identification (crosslinking and immunoprecipitation (CLIP) combined with high-throughput sequencing), bioinformatic tools and interactome capture coupled with mass spectrometry, this number is now above 1500, including conventional and non-conventional RBPs. Conventional RBPs binds to RNA targets by one or several canonical RNA-binding domains. Binding modes of non-conventional proteins to RNA are new and involve intrinsically disordered regions. 

These IDRs, through their structural flexibility, fulfill versatile roles in the constitution and remodeling of many macromolecular complexes and ultrastructures associated with mRNA throughout their life-cycle, from their biogenesis to their catabolism. IDRs also seem to be the key players during transcriptional and post-transcriptional regulation associated with all major basic processes of the cell.

**Figure 6:**
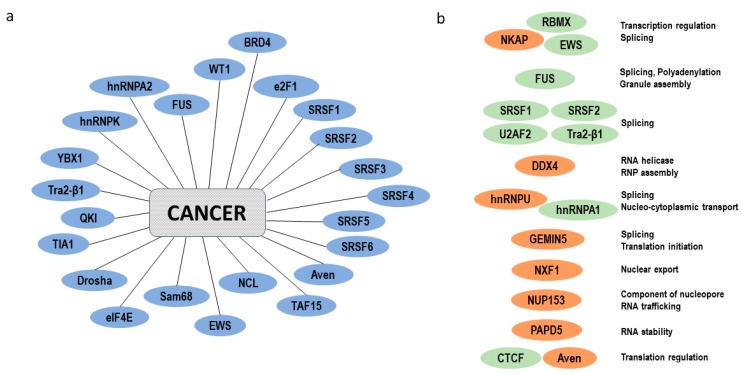
IDR-containing RBPs in mRNA life-cycle and cancer. (**a**) IDR-containing RBPs are mis-regulated in cancer cells. RBPs with IDRs may be predicted using computational analyses. The highly disordered state of the RBPs associated with cancer raises the question of whether IDRs contribute to the pathological behavior of RBPs in carcinogenesis. Some examples (from references 2 and 25) are shown. In most cases, their precise role in carcinogenesis still needs to be clarified. (**b**) IDRs-containing RBPS are involved in the whole mRNA life-cycle. The structural pliability of IDRs in proteins makes them able to interact with many different binding partners i.e., proteins, DNA and RNA. Certain disordered sequences have intrinsic RNA-binding activity, such as the RGG/RG motif. IDRs-containing RBPs are shown (examples from reference 26). Some of them contain both canonical RNA-binding domains and intrinsically disordered regions (green). Others lack any canonical RNA-binding domain but are involved in direct RNA-binding (orange). RBPs with IDRs play a role in all steps of the mRNA life-cycle.

The continuity all along mRNA life-cycle is ensured by the RBPs which provide a quick and appropriate cell response to stimuli. The major regulatory role of RBPs, and in particular of these new unconventional RBPs, raises the question of their involvement in the development of pathological processes such as carcinogenesis (Figure 6a,b). 

They thus constitute emerging or potential therapeutic targets. Their deregulation in terms of overexpression or loss of expression during carcinogenesis is an interesting, but not sufficient element, to select them as relevant targets. To achieve this purpose, investigation of their biological role and identification of their partners through cellular and animal models is a prerequisite.

## Abbreviations

aa	Amino acids
APA	Alternative polyadenylation
ARE	AU rich element
AREBP	ARE-binding protein
ASOs	Anti-sense oligonucleotides
CARE	CA repeat element
CBC	Cap-binding complex
CRD	Carbohydrate recognition domain
hnRNPs	Heterogeneous nuclear ribonucleoproteins
HuR	Human antigen R
IDR	Intrinsically Disordered Region
LC	Low complexity disordered region
miR	MicroRNA
mRBP	mRNA-binding protein
NMD	Nonsense mediated decay
NPC	Nuclear pore complex
Nt	Nucleotides
PABP	poly(A) binding protein
PBs	Processing-Bodies
PT	post-transcriptional
RBD	RNA-binding domain
RBP	RNA-binding protein
RNP	Ribonucleprotein
SGs	Stress granules
SR proteins	Serine arginine rich proteins
TTP	Tristetrapolin
UTR	Untranslated Transcribed Region
